# Analysis of weekend effect in severe acute liver injury: A nationwide database study

**DOI:** 10.1002/hsr2.139

**Published:** 2019-11-22

**Authors:** Albert Do, Ysabel C. Ilagan‐Ying, Tamar H. Taddei

**Affiliations:** ^1^ Section of Digestive Diseases Department of Internal Medicine Yale School of Medicine New Haven Connecticut USA; ^2^ Yale School of Medicine New Haven Connecticut USA

**Keywords:** acute liver failure, health services, inpatient, mortality, nationwide/national inpatient sample, predictors

## Abstract

**Background and Aims:**

Severe acute liver injury (ALI) can lead to poor outcomes without timely management. Comparatively worse outcomes in various severe, emergent conditions have been attributed to reduced hospital resources experienced by patient weekend admissions, a phenomenon termed “weekend effect.” To date, a weekend effect has not been studied in severe ALI, an emergency also necessitating timely management. We aimed to evaluate such an effect in this condition by analyzing a large national inpatient database in the United States.

**Methods:**

We analyzed the Nationwide/National Inpatient Sample (NIS) 2000 to 2014, the largest inpatient, all‐payer database in the United States (US), containing sociodemographic, clinical, patient‐, and hospital‐level data. We identified severe ALI using International Classification of Disease, 9^th^ Revision diagnosis codes for acute/subacute hepatic necrosis (570) with encephalopathy (572.2). Our primary outcome was in‐hospital mortality. Using a full‐model approach for covariate selection, we performed multiple logistic regression modeling to assess for weekend effect and identify predictors of in‐hospital mortality.

**Results:**

We identified 15 762 eligible hospitalizations, with 12 182 (77.3%) having complete covariate data. This sample comprised 53.3% males, 69.3% White race, and had an average (± SD) age of 55.0 ± 14.1 years. We utilized a full‐model approach for covariate inclusion but did not include patient transfer data due to limited availability. We observed no significant mortality differences in weekend admissions (OR = 1.06, 95% CI: 0.97‐1.15, *P* = 0.02). However, significantly higher mortality was associated with male sex, older age, Black or Hispanic race, Northeast US hospitalization, urban teaching status, and larger hospital size. Sensitivity analyses using multiple imputation datasets and transfer covariates did not change our results.

**Conclusion:**

We did not observe a weekend effect of in‐hospital mortality for weekend admissions for severe ALI, but our overall diagnosis ascertainment yield was low—indicating that lack of accurate documentation for the etiology of severe ALI may be masking an effect. Additionally, our findings suggest that racial differences and hospital‐level characteristics in the context of severe ALI may be associated with varying outcomes, regardless of admission day, which warrants further research.

**Abbreviations**
ALI: acute liver injury
CI: confidence interval
GI: gastrointestinal
HCUP: Healthcare Cost and Utilization Project
ICD‐9/10: International Classification of Diseases, 9^th^/10^th^ revision
NIS: Nationwide/National Inpatient Sample
OR: odds ratio

## 1 INTRODUCTION

Severe acute liver injury (ALI) and acute liver failure result in high morbidity and mortality in the absence of prompt diagnosis and cause‐specific management.^1^, ^2^, ^3^ Acute liver failure causes diffuse cerebral dysfunction due to cerebral edema and elevated intracranial pressure, causing high neurological morbidity and mortality in otherwise healthy adults despite potential reversibility with prompt treatment.^4^ Acetaminophen toxicity, whether by intentional or unintentional overdose, is responsible for 46% of all acute liver failure cases in the United States, and disease severity is potentially reversible with N‐acetylcysteine provision.^5^ Severe cases of Budd‐Chiari syndrome, although rare, require prompt intervention and liver transplantation.^6^ For patients with severe ALI, adverse outcomes may occur without prompt recognition and diagnosis.

Recent studies suggest that differences in patient outcomes may be associated with weekend admission, a phenomenon termed “weekend effect.” A weekend effect has been reported in the United States and Europe for a variety of severe, emergent conditions requiring prompt diagnosis and intervention, such as peptic ulcer disease‐related gastrointestinal (GI) hemorrhage, GI cancers, myocardial infarction, and stroke.^7^, ^8^, ^9^, ^10^, ^11^ Meta‐analysis for all‐cause weekend versus weekday mortality with subgroup analysis by personnel staffing levels, procedure rates, times, and delays, and illness severity, has shown that patients admitted on the weekends have a consistently higher relative risk of mortality.^12^ Several predictors for poor outcomes related to weekend admissions have been reported. Transfer status on the weekend for emergent cases like abdominal aortic aneurysm rupture may be associated with higher mortality than weekday transfers.^13^ Inconsistency in quality and access to care on the weekends^14^ and acute changes in patient status occurring on weekends requiring procedural intervention^15^ have been proposed mechanisms for this weekend effect. For ALI, these processes may also contribute to differential patient outcomes.

To date, there has been no investigation for the possibility of a weekend effect for hospitalizations with severe ALI. Severe liver injury and failure with delay in provision of cause‐specific treatment or liver transplantation may lead to poor outcomes. As ALI is an acute or subacute disease necessitating specific recognition, subspecialty management, and consideration of transfer for advanced therapies (such as liver transplantation), patients admitted on weekends may receive lower‐quality care. To study this question, we analyzed a large, national inpatient database to assess if there is evidence of an effect of weekend admission for in‐hospital mortality for severe ALI.

## 2 METHODS

### 2.1 Database

We utilized the Nationwide/National Inpatient Sample (NIS), the largest publicly available all‐payer inpatient database in the United States from the Healthcare Cost and Utilization Project (HCUP), a Federal‐State‐Industry partnership (Agency for Healthcare Research and Quality, Rockville, MD), from 2000 to 2014. Fourteen years of deidentified data were obtained, with each year of NIS containing data from an estimated 8 million hospital stays from over 1000 hospitals in 35 states, corresponding to an approximate 20% stratified sample of US community hospitals. This database contains the individual hospitalization as the unit of observation and includes sociodemographic (sex, race, income, and insurance status), clinical (length of stay, ICD‐9‐CM codes for up to 25 diagnoses), and patient‐level outcomes data (mortality, liver transplant status, and hospital transfer status). The NIS also contains hospital characteristics such as teaching status, region and urban/rural location, and hospital bed size. This database is available for purchase through the HCUP. The authors were adherent to the HCUP formal data use agreement guidelines.

### 2.2 Patient confidentiality and institutional board review

To protect patient confidentiality, the NIS does not provide identifiers that link hospitalizations to unique individuals. Thus, individual patient consent was not sought for this analysis. The Yale School of Medicine (New Haven, CT) Institutional Review Board deemed that this study, using secondary data from a public, de‐identified database, met criteria for exemption.

### 2.3 Patient sample selection, outcomes, and covariates

We included patient hospitalizations with severe ALI, defined as having International Classification of Disease, Clinical Modification, 9^th^ revision (ICD‐CM‐9) diagnosis codes of acute and subacute necrosis of liver (570) with encephalopathy (572.2). We extracted sociodemographic and clinical characteristics that may have otherwise confounded the effect of weekend admission on mortality including age, sex, race (White, Black, Hispanic, Asian, Native American, or Other), income quartile, and insurance payer (Medicare, Medicaid, Private, Other). We studied liver‐related characteristics that may also affect mortality rates, such as liver disease etiology, if known, and liver transplantation. Comorbidity burden was quantified through calculation of the Elixhauser Comorbidity Index.^16^ The primary exposure of interest was admission on a weekend day, and the outcome of interest was in‐hospital mortality. We also assessed length of stay as a secondary outcome. Both exposure (weekend admission) and outcomes (in‐hospital mortality and length of stay) were available as NIS data elements. Hospital‐related characteristics included for analysis were geographic region (Northeast, Midwest, South, West) and teaching status (rural, urban nonteaching, urban teaching).

### 2.4 Statistical analysis

Descriptive statistics on patient hospitalizations and hospital characteristics were extracted. Normality assessment of continuous variables (age) was made based on graphical assessment of the distribution histogram. Tests for differences between normally distributed variables were performed using Student's t‐test, and between non‐normally‐distributed variables, with Kruskal‐Wallis test. Differences between categorical variables were tested with chi‐square tests. We subsequently performed complete‐case univariable and multiple logistic regression modeling of in‐hospital mortality by weekend admission and other covariates. All studied covariates were included in the final model (full‐model approach) except for patient transfer status due to limited availability, as this data element was only available in the 2008 to 2014 datasets. To account for bias due to confounding by missing data or patient transfer status, we conducted additional sensitivity analyses by producing separate multiple regression models which also included: (a) patient transfer data (indicator for patients transferred in from another health facility) to account for potential confounding, and (b) 10 multiple imputation datasets to account for missing covariate data. Multiple imputation was performed using the MI command of Stata 14.2 statistical analysis software (College Station, TX, USA), utilizing fully conditional specification as to not require assumption of multivariate normal distribution.^17^ Statistical significance was defined as a two‐tailed *P* value of <.05. All analyses were performed on Stata 14.2.

## 3 RESULTS

### 3.1 Sample characteristics

From 2000 to 2014, 15 762 hospitalizations met the inclusion criteria, of which 12 182 (77.3%) had complete data. Of the 3580 hospitalizations with missing data, 3151 (88.0%) were missing data on one covariate. In the overall sample, weekend admissions were observed in 3732 (23.7%) hospitalizations. The full sample comprised 53.3% males, 69.3% of whom were White, with an average age of 55.0 ± 14.1 years (Table 1).

**Table 1:** Characteristics of patients with severe acute liver injury by admission day

	N	In Overall Sample	In Weekday Admission (n = 12 030)	In Weekend Admission (n = 3732)	*P* Value
Male Sex	15 762	53.3	53.4	52.8	0.52
Age (years)^a^	15 762	55.0 ± 14.1	55.1 ± 14.0	54.4 ± 14.2	0.008
Race	13 704				0.27
White		69.3	69.6	68.4	
Black		12.4	12.5	12.0	
Hispanic		11.3	11.0	12.5	
Asian		2.2	2.2	2.3	
Native American		1.5	1.5	1.5	
Other		3.4	3.4	3.3	
Income quartile	15 346				0.39
1^st^		28.9	28.9	29.0	
2^nd^		26.7	26.9	25.9	
3^rd^		24.2	24.0	25.2	
4^th^		20.2	20.3	19.9	
Insurance	15 715				0.11
Medicare		34.8	35.0	34.2	
Medicaid		20.5	20.4	20.8	
Private		30.5	30.7	29.7	
Other		14.2	13.9	15.4	
Etiology	15 762				0.24
Viral hepatitis		0.6	0.6	0.5	
Toxin‐induced		11.8	11.4	12.8	
Vascular		0.4	0.4	0.4	
Pregnancy		0.1	0.1	0.2	
Metabolic		1.6	1.7	1.5	
Unknown		85.5	85.8	84.7	
Elixhauser comorbidity index^b^	14 450	5 (4, 6)	5 (4, 6)	5 (4, 6)	0.50
Hospital region	15 762				0.08
Northeast		19.2	19.0	19.8	
Midwest		20.9	21.3	19.4	
South		35.6	35.4	36.2	
West		24.4	24.3	24.7	
Hospital teaching status	15 666				0.70
Rural		7.5	7.6	7.2	
Urban, non‐teaching		34.1	34.0	34.3	
Urban, teaching		58.4	58.4	58.5	
Hospital bed size	15 666				0.005
Small		10.3	10.8	8.9	
Medium		23.1	23.1	22.9	
Large		66.6	66.1	68.2	
Liver transplant	15 762	2.2	2.2	2.1	0.69
Palliative care consultation	15 762	9.9	10.1	9.5	0.30
In‐hospital mortality	15 762	42.5	42.3	42.9	0.53
Length of stay (days)^b^	15 762	8 (4, 16)	9 (4, 16)	8 (4, 15)	0.0002

All variables reported as percentage (%) unless otherwise specified; *P* values calculated with chi‐square test

a Age reported as mean ± standard deviation; *P* value calculated with Student's t‐test

b Elixhauser Comorbidity Index and Length of Stay reported as median (interquartile range); *P* value calculated with Kruskal‐Wallis test

### 3.2 Descriptive data

Overall, demographic characteristics for severe ALI hospitalizations were similar regardless of weekend or weekday status (Table 1). There were no significant differences in race, income quartile, or types of insurance. In patient care, there were no significant differences in liver transplant status or palliative care consultations. However, there was a small but statistically significant difference in age among hospitalizations with weekend admissions (54.4 ± 14.2 vs 55.1 ± 14.0 years, t‐test *P* value = 0.008) and a slightly higher proportion of hospitalizations at large hospitals (68.2% vs 66.1%, chi‐square *P* value = 0.005). In‐hospital mortality was not significantly different between groups (42.3% weekday vs 42.9% weekend, chi‐square There were inconsistencies in how this was presented throughout the manuscript.Sometimes, this was presented as "*P* value =", sometimes as "*p*", and sometimes as "*P*". Please be consistent and follow the selected reporting/typesetting guidelines.P value = 0.53). Median length of hospital stay was marginally shorter for weekend compared with weekday admissions (8 vs 9 days, respectively; Kruskal‐Wallis *P* value = 0.0002).

We additionally evaluated the 3580 patients with any missing covariate data (Supplemental Table 1). Patients excluded from the analysis were similar in proportion of weekend admissions (24.0 vs 23.6%), mortality rate (43.0 vs 42.6%), female sex (47.5 vs 46.5%), ALI etiology, and proportion receiving liver transplantation (2.5 vs 2.1%) compared with included patients, respectively (*P* value > 0.05). However, statistically significant differences were noted between excluded and included patients in age (53.6 vs 55.4 years), race (66.7 vs 69.6 White race), income quartiles (30.1 vs 28.6% first quartile), insurance provider (30.1 vs 28.6% Medicare), Elixhauser comorbidity index (median 4 vs 5), hospital region (32.9 vs 17.3% Midwest), and hospital teaching status (10.0 vs 6.8% rural), respectively (*P* value < 0.001). Additionally, length of stay was shorter in excluded patients (median 8 vs 9 days, *P* value = 0.01, Kruskal‐Wallis test).

### 3.3 Multiple logistic regression

In the final multiple logistic regression model (Table 2) including all covariates, weekend admission was not significantly associated with higher mortality (OR = 1.05, 95% CI: 0.97‐1.15, *P* = 0.22). However, older age (OR = 1.02 for each year, 95% CI: 1.01‐1.02, *P* < 0.001), male sex (OR = 1.13, 95% CI: 1.05‐1.22, *P* < 0.001), and Black or Hispanic race (OR = 1.29, 95% CI: 1.14‐1.45 and OR = 1.19, 95% CI:1.05‐1.34, respectively; *P* < 0.001) were significantly associated with mortality. In‐hospital mortality odds by toxin‐induced liver injury was significantly better than viral hepatitis‐related liver injury (OR = 0.42, 95% CI: 0.26‐0.70, *P* < 0.001). Higher comorbidity index (OR = 0.97, 95% CI: 0.95‐0.99, *P* < 0.001) and liver transplantation (OR = 0.25, 95% CI: 0.18‐0.36, *P* < 0.001) had significantly better outcomes. Placement of palliative care consult had a strong association with in‐hospital mortality compared with hospitalizations without a palliative care consult (OR = 3.64, 95% CI: 3.20‐4.14, *P* < 0.001).

**Table 2:** Multiple logistic regression analysis of in‐hospital mortality in severe acute liver injury

Characteristic	OR	95% Confidence Interval	*P* Value
Weekend admission	1.05	0.97‐1.15	0.22
Male sex	1.13	1.05‐1.22	0.001
Age (for every 1 year)	1.02	1.01‐1.02	<0.001
Race			<0.001
White	Ref	Ref	
Black	1.29	1.14‐1.45	
Hispanic	1.19	1.05‐1.34	
Asian	1.09	0.84‐1.40	
Native American	1.04	0.75‐1.46	
Other	1.07	0.86‐1.32	
Income quartile			0.35
1^st^	Ref	Ref	
2^nd^	0.92	0.83‐1.01	
3^rd^	0.93	0.93‐1.03	
4^th^	0.95	0.85‐1.07	
Insurance			<0.001
Private	Ref	Ref	
Medicare	0.90	0.81‐1.00	
Medicaid	1.07	0.95‐1.19	
Other	1.21	1.07‐1.37	
Etiology			<0.001
Viral hepatitis	Ref	Ref	
Toxin‐induced	0.42	0.26‐0.70	
Vascular	0.73	0.33‐1.62	
Pregnancy	0.24	0.40‐1.18	
Metabolic/immune	0.62	0.35‐1.09	
Other	0.75	0.46‐1.22	
Elixhauser Comorbidity Index	0.97	0.95‐0.99	0.001
Liver transplant	0.25	0.18‐0.36	<0.001
Hospital region			<0.02
Northeast	Ref	Ref	
Midwest	0.85	0.75‐0.97	
South	0.86	0.78‐0.96	
West	0.95	0.85‐1.07	
Hospital teaching status			<0.001
Rural	Ref	Ref	
Urban, nonteaching	1.50	1.27‐1.78	
Urban, teaching	1.80	1.53‐2.11	
Hospital bed size			<0.001
Small	Ref	Ref	
Medium	1.28	1.11‐1.47	
Large	1.44	1.27‐1.63	
Palliative care consultation	3.64	3.20‐4.14	<0.001

Abbreviations: **OR**, odds ratio.

Regarding hospital‐level characteristics, Midwest and South geographic region compared with Northeast hospital location were associated with better mortality outcomes (OR = 0.85, 95% CI: 0.75‐0.97 and OR = 0.86, 95% CI: 0.78‐0.96, respectively; *P* = 0.02). Additionally, urban (nonteaching and teaching) hospital status was associated with higher in‐hospital mortality compared with rural hospitals (OR = 1.50, 95% CI: 1.27‐1.78 and OR = 1.80, 95% CI: 1.53‐2.11, respectively; *P* < 0.001). Medium and large hospital size were also significantly associated with in‐hospital mortality (OR = 1.28, 95%CI: 1.11‐1.47 and OR = 1.44, 95% CI: 1.27‐1.63, respectively, *P* < 0.001).

### 3.4 Sensitivity analysis

Sensitivity analysis including hospital transfer among facility covariates yielded no statistically significant association between weekend admission and mortality (OR = 1.06, 95% CI: 0.94‐1.21, *P* = 0.34). A similar result was obtained when doing a pooled analysis using 10 multiply‐imputed datasets (OR = 1.04, 95% CI: 0.96‐1.11, *P* = 0.35).

## 4 DISCUSSION

In this analysis of 15 762 hospitalizations with severe ALI from 2000 to 2014, we identified 3732 admissions occurring in the weekend. With the exception of small, statistically significant differences between admission groups in age and hospital size—likely a reflection of comparing large sample sizes—, no other clinical differences were observed. Multiple logistic regression modeling revealed no mortality differences associated with weekend admission. This finding persisted after statistical covariate control, sensitivity analyses which included patient transfer covariates, and multiple imputation to account for missing data.

There are several potential explanations for this finding. Severe ALI can present variably over days and even months, unlike myocardial infarction, GI hemorrhage, or stroke, which often present as acute, life‐threatening events. An exception to this could be observed in hyperacute liver failure (onset <7 days), and although our study cannot stratify by ALI timing, this is a less‐common presentation.^18^ Otherwise, severe ALI may evolve over a protracted time course, with antecedent symptoms up to 12 to 24 weeks before presentation.^1^ Thus, the magnitude of disease progression over a few additional days due to weekend admission may not result in meaningful differences in mortality for this population.

Additionally, the resources needed to evaluate and treat severe ALI may not depend on weekday availability in the same manner as an emergent intervention for gastrointestinal bleed such as endoscopy, with equipment which may necessitate additional technicians or nursing staff. Except for liver transplantation, which occurs in a minority of patients with liver injury, the management of severe ALI is primarily medical. Specific medical therapies include antiviral drugs for viral hepatitis, steroids and other adjunctive therapies for autoimmune and alcoholic hepatitis, activated charcoal and N‐acetylcysteine for acetaminophen‐induced hepatitis, and prompt removal or reversal of any offending agents in drug‐induced liver injury.^1^ In contrast to procedural interventions, medical management is generally achievable in any hospital with trained medical and nursing staff, and medications are generally readily accessible as needed. However, procedural and surgical interventions may require additional providers, facilities, and equipment that require more time and effort to assemble. For example, weekend admissions for acute gallstone pancreatitis were found to experience greater delays in getting an endoscopic retrograde cholangiopancreatography (ERCP), with prolonged hospital stay and increased overall cost.^19^ The wide availability of medical therapies regardless of day of the week may also explain why the length of stay did not largely differ between weekday and weekend admissions, as needed resources are available to initiate timely diagnosis and management regardless of admission day.

The Acute Liver Failure Study Group reported a full recovery as the clinical outcome in 93.0% of patients with severe ALI.^3^ The observed survival in our sample was considerably lower, at 57.5%, which may be due to selection of patients with greater illness severity based on the diagnosis codes we used to define severe ALI, as well as inclusion of patients who progress to acute liver failure. Based on the high mortality rate observed in this sample, analysis with in‐hospital mortality as the main outcome of interest may necessitate an even larger sample size to detect significant differences when comparing weekend to weekday hospitalizations.

Although weekend admission was not significantly associated with mortality on this multiple regression analysis, other significantly associated patient and hospital‐level characteristics were identified. Compared with white populations, racial disparities may exist for Black and Hispanic patients admitted with severe ALI. Potential genetic and behavioral explanations for racial differences have been proposed in the context of other chronic liver diseases such as nonalcoholic fatty liver disease.^20^ However, our findings suggest that further research on racial differences in the context of severe ALI is warranted as well, irrespective of admission day.

Hospital‐level characteristics such as urban and large hospitals were associated with higher mortality, which some studies have attributed to the possibility of sicker patients being transferred to larger referral centers for specialized care.^13^ However, our subgroup analysis including transfer indicator variables in the multivariable model did not change the findings of the original model. Additionally, our statistical model controlled for comorbidity burden with the Elixhauser Comorbidity Index, yet still revealed hospital‐level factors as significantly associated with mortality. This suggests that other, non‐patient‐level factors may contribute to our findings, with considerations including availability of liver‐specific therapies (particularly liver transplantation) and medical staffing continuity of care. Further study of these identified and potentially unidentified factors is warranted to identify intervenable ways to improve care of patients with severe ALI.

This study benefits from several strengths, including its use of ICD‐9 diagnostic codes and analysis performed on widely standardized public data. Utilization of composite diagnosis codes for liver injury with a short‐term onset (acute and subacute necrosis of liver) may reduce inclusion of patients with acute‐on‐chronic liver failure compared with a single diagnostic code. Further combination with diagnosis of hepatic encephalopathy increases the likelihood of ascertainment of patients with severe ALI. Simplicity in defining admission day, statistical control for multiple patient‐ and hospital‐level characteristics, and unchanged results when including transfer status and multiple imputation datasets all contribute toward reduction in bias, thus strengthening study validity.

Our study has, however, some notable limitations. The composite case definition of severe ALI used diagnosis codes rather than a combination of historical, clinical, and laboratory criteria that would be needed for more definitive diagnosis.^21^ Because there is no validated, high‐performance definition for severe ALI or failure with diagnostic codes, this study is limited by the possibility of disease misclassification. However, we were careful to avoid labeling this sample as having acute liver failure, as we were unable to determine whether a given patient had chronic liver disease antecedent to hospitalization. We recognize that our analysis likely does include patients with acute liver failure, recognizing it as the severe phenotype of ALI or progression of severe ALI. In a study of diagnosis code performance in acute liver failure, Lo Re and colleagues found a positive predictive value of 67% using a combination of diagnosis codes; however, this only identified 3% of acute liver failure cases.^22^ Furthermore, the nature of the NIS database only reports on hospitalization‐level data and does not account for multiple hospitalizations by the same individual, although this would be a larger concern with more rare diseases with very small sample sizes. We did not perform statistical analysis using statistical weights or survey‐based analytical methods, as this study did not explore nationwide or temporal trends. Due to our focus on in‐hospital mortality and length of stay as outcomes of interest, we are not able to make observations on other important clinical outcomes. Previous studies have identified drug‐induced or acetaminophen‐induced liver injury as the most common culprits for ALF,^23^ and we suspect low ascertainment of this etiology, as toxic exposures may not be evident nor documented as a diagnosis code during hospitalization.

Confirmation of our findings with a prospective study of patients admitted with severe ALI would more definitively address issues of low ascertainment of identified etiologies of liver injury and further assess whether a weekend effect exists. Additionally, there may be other acute comorbidities associated with severe ALI such as development of liver‐related cerebral edema or overt progression to acute liver failure, which may confound the association of weekend admission with mortality.

In conclusion, despite previous studies suggesting mortality differences in other emergent medical conditions, we observed no evidence of a weekend effect on mortality in hospitalizations for severe ALI in an analysis of a large, national inpatient database. However, we found that mortality was associated with older age, male sex, race/ethnicity (Black or Hispanic), and viral‐induced hepatic disease, which warrants further study. Additionally, our analysis revealed that hospital‐level characteristics may impact outcomes of severe ALI hospitalizations, with higher mortality in larger, urban teaching hospitals and in the Northeast region of the United States. Future prospective studies of severe ALI and other liver diseases are needed to confirm these findings and may identify additional modifiable patient‐level and hospital‐level factors contributing to mortality risk. Ultimately, further studies are needed to address potential bias in hospital care and to prioritize hospital strategies to improve resource allocation for inpatient treatment of acute hepatic emergencies, irrespective of weekend admission status.

## FUNDING SOURCES

National Institutes of Health T32 DK007017‐41 (AD)

## CONFLICT OF INTEREST

The authors report no affiliation or involvement in potential conflicts of interest. The funding source had no involvement in study design, collection, analysis, interpretation of the data, writing of the report, nor the decision to submit the report for publication.

## AUTHOR CONTRIBUTIONS

Conceptualization: Albert Do

Data Curation: Albert Do, Ysabel C. Ilagan‐Ying

Formal Analysis: Albert Do, Ysabel C. Ilagan‐Ying

Methodology: Albert Do, Tamar H. Taddei

Supervision: Tamar H. Taddei

Writing—Original Draft Preparation: Albert Do, Ysabel C. Ilagan‐Ying

Writing—Review and Editing: Albert Do, Ysabel C. Ilagan‐Ying, Tamar H. Taddei

All authors have read and approved the final version of the manuscript. Albert Do had full access to all of the data in this study and takes complete responsibility for the integrity of the data and the accuracy of the data analysis.

## TRANSPARENCY STATEMENT

The lead author (Albert Do) affirms that this manuscript is an honest, accurate, and transparent account of the study being reported; that no important aspects of the study have been omitted; and that any discrepancies form the study as planned (and, if relevant, registered) have been explained.

## Supporting information

Supplemental Table 1.Characteristics of patients hospitalized with severe acute liver injury, stratified by study inclusion/exclusion

## Data Availability

The authors confirm that the data supporting the findings of this study are available within the article or its supplementary materials, with original source material from the appropriately cited database.
