# Supplementary material for: Analysis of weekend effect in severe acute liver injury: A nationwide database study
Source: Health Sci Rep. 2019 Nov 22;3(1):e139. doi: 10.1002/hsr2.139 (PMC7060892; doi:10.1002/hsr2.139)
Supplement: Supplementary file 1 — Supplemental Table 1.Characteristics of patients hospitalized with severe acute liver injury, stratified by study inclusion/exclusion [file HSR2-3-e139-s001.docx]

Supplemental Table 1. Characteristics of Patients Hospitalized with Severe Acute Liver Injury, stratified by study inclusion/exclusion

| **Characteristic** | **Included (n=12,182)** | **Excluded**  **(n = 3,580)** | **p-value** |
| --- | --- | --- | --- |
| Weekend Admission, % | 23.6 | 24.0 | 0.61 |
| Mortality, % | 42.3 | 43.0 | 0.51 |
| Female Gender, % | 46.5 | 47.5 | 0.30 |
| Age, mean (SD) | 55.4 ± 14.1 | 53.6 ± 13.8 | <0.001 |
| Race, %  White  Black  Hispanic  Asian  Native American  Other | 69.6  12.4  11.3  2.2  1.4  3.2 | 66.7  12.4  11.6  2.1  2.5  4.7 | <0.001 |
| Income Quartile, %  1^st^  2^nd^  3^rd^  4^th^ | 28.6  26.2  24.4  20.8 | 30.1  28.6  23.5  17.8 | <0.001 |
| Insurance, %  Medicare  Medicaid  Private  Other | 35.7  20.2  30.0  14.1 | 31,7  21.4  32.3  14.6 | <0.001 |
| Etiology, %  Viral hepatitis  Toxin-induced  Vascular  Pregnancy  Metabolic  Unknown | 0.5  9.7  0.3  0.1  4.2  85.2 | 0.4  10.3  0.4  0.2  4.1  84.7 | 0.18 |
| Elixhauser Comorbidity Index, median (IQR) | 5 (4, 6) | 4 (3, 5) | <0.001 |
| Liver transplant, % | 2.1 | 2.5 | 0.23 |
| Hospital Region, %  Northeast  Midwest  South  West | 20.8  17.3  37.3  24.6 | 13.6  32.9  29.9  23.6 | <0.001 |
| Hospital Teaching Status, %  Rural  Urban, Non-Teaching  Urban, Teaching | 6.8  35.0  58.2 | 10.0  30.6  59.4 | <0.001 |
| Hospital Bed Size, %  Small  Medium  Large | 10.7  24.0  65.3 | 9.2  19.7  71.2 | <0.001 |
| Palliative Care Consultation, % | 10.8 | 7.1 | <0.001 |
| Length of Stay (days) | 9 (4, 16) | 8 (4, 15) | 0.01 |

All variables reported as percentage (%) unless otherwise specified; p-values calculated with chi-square test

^*^Age reported as mean ± standard deviation; p-value calculated with Student’s t-test

^±^Elixhauser Comorbidity Index and Length of Stay reported as median (interquartile range); p-value calculated with Kruskal-Wallis test
